# Scalp Pruritus: Review of the Pathogenesis, Diagnosis, and Management

**DOI:** 10.1155/2019/1268430

**Published:** 2019-01-15

**Authors:** Ploysyne Rattanakaemakorn, Poonkiat Suchonwanit

**Affiliations:** Division of Dermatology, Faculty of Medicine, Ramathibodi Hospital, Mahidol University, Bangkok, Thailand

## Abstract

Scalp pruritus is a frequent problem encountered in dermatological practice. This disorder is caused by various underlying diseases and is a diagnostic and therapeutic challenge. Scalp pruritus may be localized to the scalp or extended to other body areas. It is sometimes not only associated with skin diseases or specific skin changes, but also associated with lesions secondary to rubbing or scratching. Moreover, scalp pruritus may be difficult to diagnose and manage and may have a great impact on the quality of life of patients. It can be classified as dermatologic, neuropathic, systemic, and psychogenic scalp pruritus based on the potential underlying disease. A thorough evaluation of patients presenting with scalp pruritus is important. Taking history and performing physical examination and further investigations are essential for diagnosis. Therapeutic strategy comprises removal of the aggravating factors and appropriate treatment of the underlying condition. All treatments should be performed considering an individual approach. This review article focuses on the understanding of the pathophysiology and the diagnostic and therapeutic management of scalp pruritus.

## 1. Introduction

Pruritus, also known as itch, is an unpleasant sensation that evokes a desire to scratch [1]. It is a major and distressing symptom of various cutaneous and systemic diseases. Pruritus is one of the important symptoms in dermatological practice and has a great impact on the quality of life of patients. In the skin, several etiologic factors, such as histamine, cutaneous sensory receptors, C nerve fibers, and cytokines, are involved in the pathogenesis. Pruritus can be classified into 4 categories based on its mechanisms: prurioreceptive, neurogenic, neuropathic, and psychogenic pruritus [2]. The disorder may be acute or chronic (more than 6 weeks duration) and can occur as generalized or localized.

The scalp is one of the anatomical areas frequently manifested with pruritus. The symptom is commonly associated with various scalp conditions, such as seborrheic dermatitis and psoriasis, but it often occurs without any visible skin lesion or pruritus on other body parts [3]. Systemic disorders, particularly dermatomyositis, sometimes show that scalp pruritus is one of the important treatment-resistant symptoms [4]. This specific problem was termed in previous articles as “itchy scalp,” “scalp pruritus,” or “pruritus capitis,” depending on the authors' preference. In addition, the term “trichoknesis” was introduced to describe itching sensations, which markedly increase by touching the hair [5]. Information on scalp pruritus remains limited and quite complicated. Therefore, this disorder is challenging and requires better understanding of the clinical characteristics and underlying pathogenesis to establish effective diagnosis and therapeutic approaches. This review article aims to provide the understanding of the pathophysiology and the diagnostic and therapeutic management of scalp pruritus.

## 2. Epidemiology

Although scalp pruritus is a common problem, specific epidemiological study of its prevalence is still limited. The occurrence of this disorder is reported only in case series and epidemiological studies of other conditions [6]. The prevalence of scalp pruritus in previous literature ranges from 13 to 45%.

Hoss and Segal reported that 2 of 11 patients with scalp dysesthesia manifest scalp pruritus [7]. A study from Singapore revealed a 13% prevalence of scalp pruritus among patients with generalized idiopathic pruritus [8]. In a retrospective study of sensitive scalp involving 1,011 French subjects, itching scalp was reported in 25% of the participants [9]. Another survey study among the French population reported a 21.49% prevalence of scalp pruritus [10]. Furthermore, a large epidemiological study on the prevalence of chronic pruritus involving 2,540 people found that 44.6% of subjects have scalp pruritus [11]. A cross-sectional study of 302 geriatric patients revealed a 28% prevalence of pruritus on scalp area [12]. Among 860 hemodialysis patients, the prevalence of scalp pruritus was 43.2% [13].

## 3. Pathophysiology

The pathophysiology of scalp pruritus is rarely investigated due the complex and unclear nature of cutaneous and nervous systems. Although it is a frequent problem, its pathophysiology remains unclear. Various scalp structures and mediators are hypothesized to be involved in the pathogenic process. Therefore, determining the characteristics of scalp skin anatomy and physiology is important to better elucidate the pathogenesis of scalp pruritus.

The characteristics of scalp skin are different from those of other body parts. Scalp skin has abundant sensory innervation from the branches of the trigeminal nerve and blood vessels. It contains more hair follicles and more sebaceous glands and possesses specific normal flora, resulting in susceptibility to certain dermatological problems. The pruritus signal is generally transmitted mainly by small unmyelinated C fibers that originate from the skin via the contralateral spinothalamic tract to multiple brain areas that are responsible for sensation and emotion. Moreover, several mediators, such as histamine, tryptase, substance P, bradykinin, and opioids, play a role in the mechanisms of pruritus [14]. Interestingly, aberrant C nerve fiber function of the scalp has been discovered in previous literatures that makes the scalp a unique entity in pruritus in comparison to other body areas. Shelly and Arthur reported no itch response to cowhage spicules on the occipital scalp [15]. Rukweid et al. subsequently reported that histamine-induced itch response by using intradermal microdialysis on the scalp is less than the forearm skin [16]. Furthermore, Bin Saif et al. reported a significant insensitivity of C nerve fibers of the scalp to various experimental itch induction compared with the forearm [17]. It would be of great interest to explore the role of C nerve fiber function in various disorders that manifest scalp pruritus.

The pathophysiology of scalp pruritus can be explained using 4 major mechanisms, namely, prurioreceptive, neuropathic, neurogenic, and psychogenic, which could promote more than a single mechanism [18, 19]. Prurioreceptive pruritus occurs when the itch is initiated within the skin by various inflammatory dermatological conditions that trigger the neurological pathway in the free nerve endings and then received by the brain through the unmyelinated C fibers along the contralateral spinothalamic tract. The transmission can be inhibited by stimulating the A*δ* sensory fibers through scratching, which is directed by motor reflexes, known as the “gate control theory.” Various mediators, including histamine, serotonin, prostaglandin, acetylcholine, cytokines, opioids, and neuropeptides, participate in this mechanism [14]. Neuropathic pruritus occurs along the neurological pathway where the nervous system is damaged. It is frequently associated with numbness and tingling. This type of pruritus often occurs following conditions that cause nerve damage, such as herpes zoster, trauma, and notalgia paresthetica. Neurogenic pruritus is mediated by opioid and serotonin receptors. It basically affects the central inhibitory circuits, which are related to the mechanisms of pruritus in patients with cholestasis and chronic kidney disease. Psychogenic pruritus is commonly associated with chronic stress or psychiatric disorders, such as depression and delusion of parasitosis. Psychological factors may influence itch perception.

In addition to 4 major mechanisms, the scalp normal flora is speculated to be an important factor that is involved in the pathogenesis of scalp pruritus. Microorganisms, including* Malassezia* species,* Propionibacterium* species, and staphylococci, are commonly found on the scalp [20, 21].* Malassezia* species are identified as an aggravating factor in various pruritic skin conditions, including seborrheic dermatitis, pityrosporum folliculitis, and atopic dermatitis [22, 23]. The high number of* Malassezia *species on the scalp is believed to be the cause of seborrheic dermatitis by increasing interleukin- (IL-) 8 production level [24, 25]. Moreover, the organisms contain lipase, which hydrolyzes human sebum triglycerides into free fatty acids resulting in scalp irritation and inflammation [26–28]. Staphylococci are also hypothesized to be a factor involved in the mechanism of scalp pruritus. Staphylococcal exotoxins induce IL-31 expression [29] and serine protease activation of protease-activated receptor-2 receptor [30], which are known pathways of pruritus.

Among the components of scalp skin, the stratum corneum and sebaceous glands are important in the protection of the scalp from various factors. The human scalp is exposed to environmental hazards, such as sunlight, blow-drying, mechanical trauma, and hair care products, which cause scalp inflammation. Stratum corneum acts as a barrier to protect these exogenous factors. Defect of this layer on the scalp causes scalp scaling and inflammation. Sebaceous glands produce sebum, which transfers to hair and scalp surface, which is another scalp barrier to diseases caused by bacteria and fungi [31, 32]. Patients who are sensitive to hair dyes present lower level of scalp sebum [33].

## 4. Classification of Scalp Pruritus

Several classifications have been introduced to categorize pruritus, depending on the different etiologies and clinical manifestations [1, 34]. As previously mentioned, the characteristic of scalp skin is different from that of other body parts. The scalp comprises a complex neuroanatomy with an abundance of sensorineural organs. Therefore, the causes of scalp pruritus are also different and arise from various conditions. For differential diagnostic purposes, the classification of scalp pruritus has been proposed based on the potential underlying disease, which categorizes the condition into dermatologic, systemic, neuropathic, and psychogenic diseases [35]. In addition, the term “mixed” is used when more than one underlying disease is identified. “Pruritus of undetermined origin” is used when no underlying disease can be identified [1, 36]. The proposed clinical classification of scalp pruritus is summarized in Table 1.

### 4.1. Dermatologic Conditions

Among the inflammatory dermatoses, seborrheic dermatitis is the most common disorder presented with scalp pruritus [20]. The clinical presentation is erythematous patches or plaques with scales on the scalp. Dandruff, known as pityriasis capitis, is considered a mild form of seborrheic dermatitis, which presents scalp scaling without erythema. The pathogenesis of seborrheic dermatitis is mediated from interactions among scalp skin, sebum,* Malassezia* fungi, and the immune system [26]. The histamine level in the scalp skin is high in patients with seborrheic dermatitis. Reduction of histamine levels is associated with a significant reduction in the intensity of pruritus [37]. Scalp psoriasis is another inflammatory skin disease that commonly presents with scalp pruritus. The scalp is the most affected area in psoriasis [38]. A survey study of 195 patients with psoriasis revealed that 58% of patients report scalp pruritus [39]. Other inflammatory dermatoses that are associated with scalp pruritus are atopic dermatitis [39], contact dermatitis [9], pityriasis amiantacea [40], and angiolymphoid hyperplasia with eosinophilia [41].

Scalp pruritus is also commonly reported in patients with cicatricial alopecia when inflammation occurs. Lichen planopilaris (LPP) [42–44] and frontal fibrosing alopecia, variant of LPP [45], were reported in approximately 70% and 67% of patients with scalp pruritus, respectively. Central centrifugal cicatricial alopecia (CCCA), a common cause of scarring alopecia in African American women, is frequently associated with scalp pruritus [46, 47]. An experimental study in patients with CCCA revealed the association of disease severity with cowhage-induced itch [48]. Folliculitis decalvans, a neutrophilic cicatricial alopecia, is also present with scalp pruritus [49].

Other dermatologic conditions, such as infectious dermatoses, autoimmune dermatoses, and neoplasms, can present with scalp pruritus. The common infectious diseases are pediculosis capitis and scabies that often occur in children. Cutaneous larva migrans of the scalp was also reported in literature [50]. Red scalp disease, an unknown etiologic condition, presents a diffuse erythema of the scalp with pruritus [51].

### 4.2. Neuropathic Conditions

Pathologic involvement of small unmyelinated C nerve fibers is an important mechanism in neuropathic conditions. Neuropathic pruritus of the scalp is commonly associated with diabetes mellitus and herpes zoster. Scribner revealed that several patients who present scalp pruritus achieve a complete relief when the underlying diabetes is controlled [52]. Postherpetic neuralgia (PHN) following herpes zoster was also reported to be associated with pruritus. Scalp pruritus is more likely to develop after herpes zoster, particularly in the trigeminal (V1) dermatome [53, 54]. A retrospective study of 600 patients with PHN revealed that scalp pruritus often occurs after herpes zoster on the head, face, and neck [54]. Other neuropathic conditions that were reported to be associated with scalp pruritus are scalp dysesthesia [7], brain and spinal cord injury [55], narrowing of the bony foramina from osteoarthritis [55], Wallenberg syndrome [56], and brain tumors [57].

### 4.3. Systemic Conditions

Systemic diseases, including chronic kidney disease, cholestatic liver diseases, and hematologic malignancy, are associated with chronic pruritus. The scalp is also one of the common parts complained by patients with systemic diseases. Other systemic conditions reported are drug-induced pruritus by dobutamine [58] and eosinophilic arteritis of the scalp [59].

Scalp involvement in dermatomyositis is common, and patients can present with scalp pruritus [60, 61]. Recently, scalp pruritus in dermatomyositis has been associated with small-fiber neuropathy. A confocal image of immunostained scalp skin biopsies from a case of dermatomyositis with severe scalp pruritus revealed decreased density and formed complex tufts of epidermal nerves, which could explain the mechanism of pruritus in patients with dermatomyositis [62].

### 4.4. Psychogenic Conditions

Psychogenic factors, such as anxiety, depression, and delusion of parasitosis, can be the cause of scalp pruritus. In any cases, the scalp is a frequent area of excoriations, possibly because this area is easily accessible. In patients with generalized pruritus, more than 10% are triggered by psychological factors [63]. Ferm et al. reported that the most commonly affected parts in patients with psychogenic pruritus are the scalp and face. Their retrospective study of patients with chronic pruritus showed that 16.7% of patients with psychiatric disease had pruritus of the scalp, which was also the most frequent affected area reported among psychiatric patients [64].

In 2013, Reich et al. introduced the term “trichoknesis” to describe itching sensations, which markedly increase by touching the hair [5]. Trichoknesis is considered a variant of trichodynia, a somatoform disorder, which is a painful sensation within hair that becomes more intense when hairs are touched. However, since Ericson et al. discovered localization of substance P in the scalp of patients with trichodynia [65], suggesting a causal relationship, its pathophysiology is probably polyetiologic [66]. In addition, small-fiber neuropathy, an alteration of the nervous system, was hypothesized to be one of the pathogenic processes of both trichodynia and trichoknesis [67].

### 4.5. Scalp Pruritus of Undetermined Origin

Scalp pruritus with no underlying condition following completion of evaluation is difficult to diagnose. Physicians often discuss with their patients and tell them that their scalp is sensitive. Sensitive skin is characterized by complaints of discomfort sensation, such as itching, burning, and stinging without predictable clinical signs [68, 69]. In this condition, the skin decreases its tolerance threshold against various triggering factors with no associated skin diseases [70, 71]. Scalp is one of the common affected areas of sensitive skin, the so called “sensitive scalp.” Previous studies reported 32 to 44% incidence of sensitive scalp [9, 72]. A study from French population has identified the triggering factors of sensitive scalp, including heat, cold, pollution, emotions, dry air, wet air, water, and shampoos [9]. In addition, hair dyeing products have been demonstrated to play a role in increasing scalp sensitivity [73]. Scalp pruritus was reported as a symptom in 60% of patients with sensitive scalp; it was found more in women than men and increased with age [70]. However, the pathophysiology of sensitive scalp remains unclear. Categorizing the sensitive scalp into the proposed classification is difficult, and the term “scalp pruritus of undetermined origin” is suitable.

## 5. Diagnostic Approach of Scalp Pruritus

The initial diagnostic approach in patients with scalp pruritus includes history taking and physical examination to determine whether the scalp pruritus is caused by a dermatologic condition or is secondary to other underlying conditions. The presence of a primary skin lesion suggests that evaluation should focus on a dermatologic cause. In the absence of a primary skin lesion, systemic conditions should be included in the evaluation process.

### 5.1. History Taking

The history taking for evaluating scalp pruritus should include information on the onset, affected areas other than the scalp, type, severity, and aggravating and alleviating factors. A detailed history should focus on recent exposures to new possible aggravating factors, such as hair care products (e.g., shampoos, conditioners, hair styling aids, hair dyes, cosmetics applied to the scalp), hair care practice, and current medications. Environmental factors, including sun exposure, travel history, and occupational exposure, are also important to explore. History of contact with people or animals with risk of exposure to infectious organisms, such as tinea capitis, pediculosis capitis, and scabies, is also important. In addition, the review of systems for underlying systemic disease should be performed, particularly for atopy, diabetes mellitus, thyroid diseases, hematologic malignancy, chronic kidney disease, and cholestatic liver diseases. Patients with neuropathic scalp pruritus often present abnormal sensations, such as burning and stinging, along with pruritus. Currently, no standardized method has been established to evaluate the intensity of scalp pruritus. A visual analogue scale is a useful method to indicate pruritus intensity on a line, from 0 (no pruritus) to 10 (the worst pruritus) [74].

### 5.2. Physical Examination

Physical examination should focus on the dermatological system, particularly the hair and scalp area, for evidence of any recognizable skin conditions. Establishing whether scalp pruritus preceded the appearance of a skin condition is important. Primary and secondary skin lesions need to be discriminated. Primary skin lesions originate from an underlying condition and are important for evaluating the dermatological cause. Manipulations, such as scratching and rubbing, can lead to secondary skin lesions, such as excoriation and lichenification, providing a chronic course of the disease. Secondary skin lesions may resolve, leaving dyspigmentation and scars. Moreover, evidence of infection, particularly tinea capitis, pediculosis capitis, and scabies, should be examined. Bedside examinations, such as dermoscopy, examination using a Wood's lamp, and microscopic examination, should be performed to help provide definite diagnosis. Although pruritus of systemic disease is often generalized, it may sometimes present as localized pruritus. Therefore, a complete physical examination on other body parts should be performed including an evaluation of the liver, spleen, and lymph nodes. Neurological system evaluation is necessary in patients with suspected neuropathic scalp pruritus. Abnormalities in other systems increase the possibility of underlying systemic diseases. However, the presence of primary skin lesion does not exclude an underlying systemic disease, and the absence of primary skin lesion does not imply that the causal condition is a systemic disease.

### 5.3. Investigation

If the diagnosis remains unclear after history taking and physical examination, laboratory investigation should be performed following the findings of prior evaluation. An initial laboratory investigation consists of complete blood count, blood urea nitrogen, creatinine, bilirubin, alkaline phosphatase, thyroid-stimulating hormone, and fasting plasma glucose. Further investigations may include skin scraping and culture, skin biopsy, antinuclear antibody, anti-hepatitis C virus antibody, anti-human immunodeficiency virus antibody, and chest X-ray. In patients with suspected neuropathic scalp pruritus, further investigations, such as nerve conduction study, electromyography, and magnetic resonance imaging, may be indicated.

**Figure 1:**
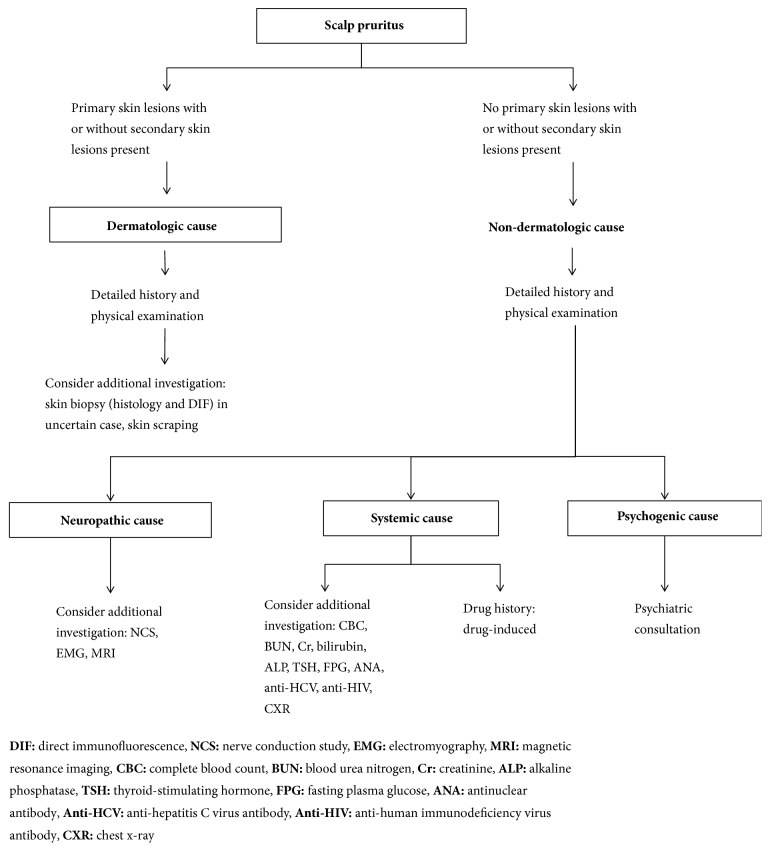
Diagnostic approach for scalp pruritus.

When all evaluations reveal negative results, patients with chronic persistent scalp pruritus should be followed up with repeated evaluation. The cause of scalp pruritus may present later. Another point to consider is the presence of underlying psychological problems, which should be addressed immediately by referring the patient to a psychiatrist.  Figure 1 outlines a diagnostic approach for patients who present with scalp pruritus.

## 6. Management

Management of scalp pruritus is challenging because it is complex, multifactorial, and no generally accepted strategy exists. The principle of treatment includes removal of the aggravating factors and appropriate treatment of the underlying condition. Topical therapy is the mainstay of treatment. For widespread and recalcitrant diseases, a therapeutic approach by combination of topical and systemic therapy is indicated. In addition, supportive therapy by other modalities is necessary to maximize therapeutic outcome. Various treatments have been introduced to treat scalp pruritus; however, the efficacy of these treatments on scalp pruritus is still limited since pathophysiology of pruritus in most disorders is unclear. Hair care practices should be performed gently by using hypoallergenic products and avoidance of chemical irritants, fragrance, and hot blow-drying. Shampoos with anti-inflammatory effects, such as zinc pyrithione, ketoconazole, selenium sulfide, and coal tar, can be used to reduce scalp inflammation. Depending on the underlying mechanism, therapeutic options used should be selected following the pathogenic process. Specific treatment of a primary condition often results in a relevant improvement of scalp pruritus. However, immediate improvement of scalp pruritus is more important and is the first step of the treatment plan.

Notably, the characteristic of scalp skin can prevent optimal treatment. The presence of hair can interfere with treatment reaching the scalp, and scalp thickness decreases the penetration. Certain vehicles, such as ointment and cream, can be messy to apply and adhere to the hair shaft, resulting in a greasy appearance and decreased patient compliance due to cosmetic unacceptability. Therefore, the optimal vehicle can be as important as the active ingredient in achieving efficacy, tolerability, and adherence. Lotion, gel, and foam are preferred vehicles owing to their superiority to cream and ointment in the treatment within the scalp area. Lack of medications in appropriate forms is also a problem in the treatment of scalp pruritus. Moreover, physicians should educate and encourage their patients to use medications regularly as prescribed to achieve treatment effectiveness.

### 6.1. Topical Therapy

An appropriate short acting method to relieve acute scalp pruritus includes moisturizing with scalp lotions and oily emollients containing glycerin or panthenol that helps scalp dryness. Use of shampoo with an optimal pH (4.5-6.0) reduces the secretion of serine proteases that can initiate scalp pruritus [75]. In addition, cooling scalp with menthol- or camphor-containing shampoo/lotion or using local anesthetics containing shampoo/lotion-like polidocanol can temporarily reduce scalp pruritus. Polidocanol, a protease-activated receptor-2 antagonist, is a nonionic surfactant with local anesthetic effect. Menthol provides antipruritic effect by generating a cool sensation via activation of transient receptor potential melastatin 8 [76, 77]. However, careful use of menthol is necessary because it can induce skin irritation. Cold compression may help relieve scalp pruritus. Studies on topical antihistamines reveal ineffectiveness and may cause allergic contact dermatitis [78].  Table 2 demonstrates a list of the principal topical medications for scalp pruritus.

Topical corticosteroids can provide a quick relief from scalp pruritus in steroid-responsive dermatosis and are available in solutions, foams, and shampoos, which are convenient to use on the scalp. However, studies providing evidence of a significant antipruritic effect in the treatment of scalp pruritus rarely exist [79]. Therefore, single application of topical corticosteroids for treating pruritus symptom is not advised. Nevertheless, prolonged use of topical steroids on scalp can induce skin atrophy.

The topical calcineurin inhibitors, tacrolimus and pimecrolimus, were introduced for the treatment of atopic dermatitis. In addition to improving skin lesions, they also show antipruritic effect. Compared with capsaicin, an initial burning and pruritus possibly occur, which may provide clinical evidence for assumed transient receptor potential vanilloid 1 (TRPV1) binding. Topical calcineurin inhibitors have been proven to be effective for various pruritic skin conditions, such as prurigo nodularis and genital chronic pruritus [80], and could be an alternative option for scalp pruritus. However, medications are only available in ointment and cream which could difficult to use on scalp area.

Capsaicin, a substance that binds to the TRPV1 receptor, is an option for the symptomatic treatment of neuropathic scalp pruritus. Topical capsaicin is available in solutions which is convenient to use on the scalp. This substance owes its antipruritic mechanism to desensitization of the sensory nerve fiber and interrupts the conduction of cutaneous pruritus. Therefore, topical capsaicin has been reported to be effective in postherpetic neuralgia, notalgia paresthetica, brachioradial pruritus, aquagenic pruritus, and prurigo nodularis [81, 82].

Liquor carbonis detergens, a tar derivative, have an anti-inflammatory and soothing effect to relieve scalp pruritus. It can be alternatively used for resistant inflammatory dermatoses and is available in 3-10% solutions and shampoos. It is often used to treat scalp seborrheic dermatitis and scalp psoriasis.

### 6.2. Systemic Therapy

Systemic therapy is rarely indicated in the treatment of scalp pruritus. However, this option should be considered when topical therapy is ineffective [83]. Currently, no study has investigated the efficacy of any systemic therapies for the treatment of scalp pruritus. However, initiation of systemic therapy is based on a step-by-step approach depending on the severity of scalp pruritus, the overall status of the patient, and expected adverse effects of the treatment. A list of the principal systemic medications for scalp pruritus is summarized in Table 3.

Antihistamines are frequently used in general practice for the treatment of various types of pruritus. Its mechanism of H1 receptor antagonists is speculated to be effective when pruritus is mediated by histamine. Antihistamines also modulate immunological responses, such as mediator release, cytokines, and chemokines. The role of antihistamines for the treatment of other pruritic disorders, other than urticaria and mastocytosis, remains controversial [84]. However, previous studies suggested that high dosage or combination of antihistamines show effectiveness in the treatment of chronic pruritus with different origins [85].

Anticonvulsants and pain modulators, such as gabapentin and carbamazepine, which are inhibitors of the neuropathic afferent pathway, are used for neuropathic pain and also have antipruritic effects [86]. The antipruritic mechanism is still unknown, but it has been speculated to involve in both central and peripheral pathways. Gabapentin inhibits release of calcitonin gene-related peptide from primary afferent neurons by increasing gamma-amino butyric acid in the spinal cord [87]. Gabapentin should be considered as a treatment option in patients with suspected neuropathic scalp pruritus; the dose of gabapentin is usually started low and titrated up to an effective dose [83]. Adverse effects reported include dizziness, peripheral edema, and worsening of diabetes mellitus.

Opioid receptor antagonists, such as naloxone and naltrexone, influence the neurogenic pruritus by inhibiting itch transmission. Opioid receptor antagonists showed antipruritic effects in cholestatic pruritus, uremic pruritus, and various pruritic skin conditions, such as atopic dermatitis, bullous pemphigoid, cutaneous lymphoma, and prurigo nodularis [88]. These antagonists should be considered as a treatment option for neuropathic scalp pruritus. Adverse effects, such as nausea and vomiting, are reported in the first few days.

Antidepressants directly influence central pruritus by unknown mechanisms. Their mechanism is speculated to interfere in the neuronal reuptake of neurotransmitters, such as serotonin and norepinephrine. Tricyclic and tetracyclic antidepressants have been reported to be effective. Amitriptyline is useful in some cases of neuropathic pruritus. Doxepin and mirtazapine possess additional antihistaminic effect [89, 90]. Selective serotonin reuptake inhibitors reveal similar effects with a better safety profile. Paroxetine has antipruritic effect in patients with various types of nondermatologic pruritus [91].

The other medications that may be considered for treatment of scalp pruritus with special caution are cyclosporin A and thalidomide. Both medications reveal antipruritic effects with serious adverse effects. Cyclosporin A is a potent immunosuppressive agent that possesses significant antipruritic effect [92]. The antipruritic mechanism is assumed to be symptomatic due to the anti-inflammatory effects. Thalidomide acts as an immunomodulatory drug, a tumor necrosis factor-alpha inhibitor, and a central and peripheral nerve depressant; it is effective in the treatment of various types of pruritus [93].

### 6.3. Phototherapy

Ultraviolet (UV) phototherapy is widely used in patients with pruritus. Both UVB and PUVA (psoralen and UVA) therapies successfully relieve the symptom. Phototherapy provides anti-inflammatory effect, antiproliferative effect, and mast-cell apoptosis with less adverse events [94]. Its efficacy in the treatment of pruritus has been reported in previous studies [95–98]. Although phototherapy has never been specifically reported in the treatment of scalp pruritus, the use of targeted phototherapy or a UV-light comb may be considered in recalcitrant cases since the devices can improve the transmission of UV to the scalp surface.

### 6.4. Psychotherapy

Psychotherapies, such as behavior therapy, need to be considered to break the vicious circle of itching and scratching [99]. Some patients show an unconscious automatic scratching behavior. The benefits of psychotherapy include stress reduction and increased sense of control of scratching [100]. More importantly, the therapeutic strategies do not harm and are likely to improve patient quality of life. In patients with underlying psychiatric disorders, psychotherapy in combination with medical therapy can be helpful to treat scalp pruritus.

## 7. Conclusion

Scalp pruritus continues to be a major dermatological problem. It is a common and sometimes disabling symptom. The diagnostic approach to patients with scalp pruritus is complicated and requires multidisciplinary interactions due to the complex neuroanatomy of the scalp and incomplete understanding of the pathogenesis. Although the understanding of the pathogenesis of scalp pruritus has improved significantly in recent years, it remains one of the great challenges for medical research. Further investigation and determining more effective antipruritic agents with lower adverse effects are necessary.

## Figures and Tables

**Table 1:** Proposed clinical classification of scalp pruritus.

**Classification**	**Associated disease**
**Dermatologic conditions**	***Inflammatory dermatoses*:** acne necrotica, alopecia areata, angiolymphoid hyperplasia with eosinophilia, atopic dermatitis, central centrifugal cicatricial alopecia, contact dermatitis, discoid lupus erythematosus, folliculitis decalvans, frontal fibrosing alopecia, insect bite, lichen planopilaris, lichen simplex chronicus, pityriasis amiantacea, psoriasis, red scalp disease, scars, seborrheic dermatitis, urticaria, xerosis ***Infectious dermatoses*:** cutaneous larva migrans, folliculitis, impetigo, pediculosis capitis, scabies, tinea capitis ***Autoimmune dermatoses*:** bullous pemphigoid, dermatitis herpetiformis ***Neoplasms*:** leukemia cutis, lymphoma cutis

**Neuropathic conditions**	Atypical facial neuralgia, brain and spinal cord injury, brain tumors, migraine headache, narrowing of the bony foramina from osteoarthritis, post herpetic neuralgia, scalp dysesthesia, Wallenberg syndrome

**Systemic conditions**	Cholestatic liver disease, chronic renal failure, dermatomyositis, diabetes mellitus, drug-induced pruritus (dobutamine), eosinophilic arteritis of the scalp, Hodgkin and non-Hodgkin lymphoma

**Psychogenic conditions**	Anxiety disorders, delusional parasitosis, depression, obsessive compulsive disorders, schizophrenia, somatoform and dissociative disorders, tactile hallucinations

**Scalp pruritus of undetermined origin**	Sensitive skin

**Table 2:** Principal topical medications for scalp pruritus.

**Medication**	**Dosage**	**Adverse effects**
**Glucocorticoids**	Many drugs with different doses; prefer using lotion, gel, and foam as vehicle	Skin atrophy, folliculitis, telangiectasia

**Calcineurin inhibitors**		
Pimecrolimus	1%	Stinging or burning sensation
Tacrolimus	0.03% - 0.1%	Stinging or burning sensation

**Miscellaneous**		
Menthol	1% - 5%	Skin irritation
Capsaicin	0.025% - 0.1%	Burning sensation
Liquor carbonis detergens	3% - 10%	Skin irritation, stinging sensation
Shampoos with anti-inflammatory effects	Shampoos containing zinc pyrithione, ketoconazole, selenium sulfide, or coal tar	Skin irritation, scalp dryness

**Table 3:** Principal systemic medications for scalp pruritus.

**Medication**	**Dosage**	**Adverse effects**
**Antihistamines**		
Chlorphenamine	4 - 16 mg/day orally	Drowsiness, dry mouth
Hydroxyzine	25 - 50 mg/day orally	Drowsiness, dry mouth
Diphenhydramine	25 - 100 mg/day orally	Drowsiness, dry mouth
Cetirizine	10 - 20 mg/day orally	Drowsiness, dry mouth
Loratadine	10 - 20 mg/day orally	Drowsiness, dry mouth
Fexofenadine	60 - 360 mg/day orally	Drowsiness, dry mouth
Levocetirizine	5 - 10 mg/day orally	Unusual drowsiness, dry mouth
Doxepin	25 - 100 mg/day orally	Drowsiness, dry mouth, prolonged QT interval

**Anticonvulsants**		
Gabapentin	100 - 1200 mg/day orally	Drowsiness, leg edema, constipation
Pregabalin	25 - 200 mg/day orally	Drowsiness, leg edema

**Opioids**		
Naloxone	0.2 mg/kg/min intravenous daily, preceded by 0.4mg intravenous bolus over 24 hours	Hepatotoxicity, nausea and vomiting, insomnia
Naltrexone	12.5 - 50 mg/day orally	Hepatotoxicity, nausea and vomiting, abdominal pain, diarrhea
Butorphanol	1 - 4 mg inhaled at bedtime	Drowsiness, nausea and vomiting, dizziness

**Antidepressants**		
Amitriptyline	10 - 150 mg/day orally	Drowsiness, dizziness, dry mouth, constipation
Paroxetine	10 - 40 mg/day orally	Insomnia, dry mouth, sexual dysfunction
Mirtazapine	7.5 - 15 mg/day orally	Drowsiness, weight gain, dry mouth

**Miscellaneous**		
Cyclosporin A	3 - 5 mg/kg/day orally	Nephrotoxicity, hypertension
Thalidomide	100 - 200 mg/day orally	Teratogenic effect, peripheral neuropathy, drowsiness, constipation
